# Regional Head-and-Neck Lymphadenopathy with Dysphagia and Dysphonia: An Airway-Threatening Presentation of HPV-16–Positive Oropharyngeal Squamous Cell Carcinoma (OPSCC)

**DOI:** 10.51894/001c.166043

**Published:** 2026-08-01

**Authors:** Zeeshan Rizvi

**Affiliations:** 1 College of Osteopathic Medicine Michigan State University

## 134

### Introduction

Human papillomavirus (HPV), particularly HPV-16, is now a leading cause of oropharyngeal squamous cell carcinoma (OPSCC), transmitted primarily through oral exposure. Despite rising incidence, clinical awareness of its variable presentations remains limited. Early recognition is essential because airway compromise may progress rapidly even when initial imaging appears benign.

### CASE DESCRIPTION

A 37-year-old woman experienced 14 months of progressive cervical lymphadenopathy, dysphagia, dysphonia, and ultimately right brachial plexopathy. She underwent eight hospitalizations without lymph node biopsy and received multiple rounds of steroids and/or antibiotics, which temporarily masked progression. She later developed stridor and presented to the emergency department where computed tomography was interpreted as negative and she was discharged. Persistent clinical concern prompted direct physician–radiologist communication and independent reinterpretation of imaging, which revealed extensive cervical lymphadenopathy with near-complete nasopharyngeal obstruction and marked lingual tonsillar enlargement compressing the soft palate. The patient returned emergently with impending airway compromise. Severe upper-airway obstruction secondary to HPV-16–positive, p16-positive OPSCC with bulky nodal disease and neurologic involvement. The patient required emergent intubation for impending airway compromise using a pediatric-sized endotracheal tube, followed by tracheotomy and four days of mechanical ventilation before definitive biopsy and diagnosis. Coordinated airway management and expedited oncologic evaluation were essential for stabilization. Histopathology confirmed HPV-16–positive squamous cell carcinoma involving the naso- and oropharynx. The patient gradually weaned from ventilatory support and recovered after approximately 11–12 days of airway stabilization. She was discharged to rehabilitation to improve functional status while awaiting PET-CT staging and initiation of definitive chemoradiation therapy.

### Discussion

This case illustrates how a siloed clinical approach, delayed biopsy, and reliance on transient steroid response can obscure a rapidly evolving malignancy. Cross-disciplinary review of patient presentations helps identify alternative pathologies and prompts earlier consideration of alternative diagnoses and management strategies. This is imperative when presentations mimic benign infection such as HPV-16. A structured approach including early consideration of HPV-related etiologies, prompt tissue diagnosis in persistent or progressive lymphadenopathy, and coordinated multidisciplinary communication may reduce diagnostic delay and prevent life-threatening airway emergencies.

